# Cardiac Autonomic Neuropathy: Why Should Cardiologists Care about That?

**DOI:** 10.1155/2017/5374176

**Published:** 2017-10-29

**Authors:** Andrzej Bissinger

**Affiliations:** Department of Interventional Cardiology and Arrhythmias, Medical University of Lodz, Lodz, Poland

## Abstract

**Background:**

Cardiac autonomic neuropathy (CAN) is a frequent but underdiagnosed complication of diabetes mellitus. It has a strong influence on various cardiac disorders including myocardial ischemia and infarction, hypertension, orthostatic hypotonia, heart failure, and arrhythmias. CAN can lead to severe morbidity and mortality and increase the risk of sudden cardiac death.

**Methods:**

This review article summarizes the latest evidence regarding the epidemiology, pathogenesis, influence on the cardiovascular system, and diagnostic methods for CAN. The methodology of this review involved analyzing available data from recent papers relevant to the topic of diabetic autonomic neuropathy and cardiac disorders.

**Conclusions:**

The early diagnosis of CAN can improve the prognosis and reduce adverse cardiac events. Methods based on heart rate variability enable the diagnosis of CAN even at a preclinical stage. These methods are simple and widely available for use in everyday clinical practice. According to the recently published Toronto Consensus Panel on Diabetic Neuropathy, all diabetic patients should be screened for CAN. Because diabetes mellitus often coexists with heart diseases and the most common methods used for diagnosis of CAN are based on ECG, not only diabetologists but also cardiologists should be responsible for diagnosis of CAN.

## 1. Introduction

Diabetes mellitus (DM) affects at least 8.5% of the global population, that is, about 422 million people worldwide [1]. Diabetes leads to complications in many parts of the body and can increase the overall risk of dying prematurely. Possible complications include heart attack, stroke, kidney failure, leg amputation, vision loss, and nerve damage. It is difficult to estimate the actual prevalence of diabetic complications, because especially microvascular complications are often underdiagnosed. The incidence of cardiac or cerebrovascular disease is two to four times higher in diabetic patients than in the general population. The leading cause of mortality and morbidity in patients with DM is cardiovascular diseases (CVD), such as coronary artery disease (CAD). The influence of diabetes on CAD is synergistic with other factors, such as age, hypercholesterolemia, hypertension, and smoking. Therefore, most preventive strategies focus on improving glycemic control, lowering blood pressure, and treating dyslipidemia [2]. Despite that CVD remains the major cause of mortality and morbidity in patients with DM, the autonomic nervous system (ANS) has an important influence on CVD. Two parts of the ANS—parasympathetic (PNS) and sympathetic (SNS)—cooperate to control heart rate, cardiac output, myocardial contractility, and constriction and dilatation of blood vessels [3]. Cardiac autonomic neuropathy has a major impact on CVD in diabetes. CAN is a very common complication of DM and very often not diagnosed. Based on the Subcommittee of the Toronto Consensus Panel on Diabetic Neuropathy, CAN is defined as the impairment of cardiovascular autonomic control in patients with established DM following the exclusion of other causes [4].

## 2. Epidemiology and Pathogenesis

There is huge variation in CAN prevalence depending on the diagnostic methods used, population studied, and disease stage. As reported in major studies, the number of patients with CAN varies from 17% to 90% in patients with DM type 1 and 27.5% to 73% in patients with DM type 2 (Table 1). The duration of diabetes is an independent factor for developing CAN irrespective of diabetes type [4, 5]. CAN is detected in about 7% of patients with DM type 1 or 2 at the time of diagnosis, and it is estimated that the risk increases annually by about 6% and 2% in patients with DM type 1 and 2, respectively [5, 6]. Other risk factors for developing CAN are poor glycemic control, age, obesity, smoking, hypertension, distal polyneuropathy, nephropathy, and retinopathy [3, 7]. Poor glycemic control is a major risk for CAN progression [7–9]. In the Diabetes Control and Complication Trial (DCCT), intensive glycemic control resulted in 50% reduction in CAN incidence over the 6.5 years of follow-up [10]. Other interventions targeting hypertension, smoking, obesity, and hyperlipidemia also decrease the incidence of CAN [2, 11]. The impact of gender on CAN is controversial. The EURODIAB IDDM Complications Study did not reveal differences in CAN frequency between men (35%) and women (37%) [12]. However, the ACCORD study showed that CAN was more prevalent in women (2.6% of men versus 4.7% of women, *p* < 0.01) [13].

CAN is caused by complex interactions and involves several mechanisms and pathways that lead to neuronal ischemia and finally neuronal death [8, 9]. The leading cause of the pathogenic process is hyperglycemia [3, 5]. Hyperglycemia-induced oxidative stress and toxic advanced glycosylation products lead to changes in mitochondrial functions, membrane permeability, and endothelial functions. These different pathways induce changes in gene expression, transcription factors, disruption of several cellular functions, and communication between the cells and surrounding matrix. All of this leads to neuronal dysfunction and death [9, 14].

The early stages of CAN damage the vagus nerve, thus leading to sympathetic predominance. This increase in sympathetic tone continues until advanced CAN, when sympathetic denervation also ensues [9].

Recent papers have also reported an association between hypoglycemia and the autonomic nervous system. Cichosz et al. found that the HRV parameter low frequency (LF) was significantly reduced during hypoglycemic episodes in patients with and without CAN [15]. Jaiswal et al. found that hypoglycemic stress reduced HRV power independently of glucose control as assessed by HbA1c [16]. These data suggest that not only hyperglycemia but also high glucose variability may contribute to CAN.

## 3. Clinical Roles of CAN in Cardiology

### 3.1. Coronary Artery Disease

Coronary artery disease (CAD) is a major complication of DM. The most typical clinical evidence of CAN in patients with concomitant CAD is silent myocardial ischemia (SMI) [17]. A meta-analysis of 12 studies showed that CAN is associated with SMI detected by the exercise test with prevalence ratios of 0.85 to 15.53 (the Mantel-Haenszel estimate for prevalence rate risk was 1.96, 95% CI: 1.53–2.51, *p* < 0.001) [6]. Several publications have reported the poor clinical outcome of patients with SMI. A threefold increase in cardiac deaths was witnessed over a 2-year follow-up in individuals with SMI detected during Holter ECG [18]. The Framingham Heart Study showed significantly higher incidence of painless myocardial infarction in patients with DM than without DM (39% versus 22%) [19]. A study of 120 patients with DM and no previous CAD found that CAN was a better predictor of major cardiovascular events (such as sudden death, death caused by MI, congestive heart failure, nonfatal MI, heart failure, resuscitation from ventricular tachycardia/fibrillation, and need for coronary revascularization) than the presence of SMI (OR = 4.16, 95% CI: 1.01–17.19) and when CAN was combined with SMI the risk was even higher (5 out of 10 had a major event). CAN was also found to be associated with higher mortality risk in patients after myocardial infarction [20]. Features of myocardial infarction in patients with CAN may include dyspnea, fatigue, heart palpitations, hypotonia, nausea, and vomiting [21]. Although CAN might be used to stratify coronary artery risk and the screening of coronary artery disease might be beneficial in patients with CAN, there is no consensus on this point. The point of cost-effectiveness of this strategy has not been proven yet. After myocardial infarction, screening for CAN can be used for further risk stratification.

Autonomic dysfunction, assessed as reduced heart rate variability, was also associated with coronary artery calcification [22, 23]. Whether autonomic dysfunction is involved in the pathogenesis of atherosclerosis itself, it would have important implications for our understanding of the pathogenesis of coronary atherosclerosis in diabetic patients. A direct effect of autonomic dysfunction on atherosclerosis is certainly plausible. Sympathetic denervation may cause dedifferentiation of vascular smooth muscle cells and alteration to a phenotype associated with extracellular matrix production and migration to the intima, changes that have been observed in atherosclerosis [24, 25]. An important issue is whether prevention of CAN might confer the added benefit of reduced coronary artery disease in diabetic patients.

### 3.2. Hypertension

Hypertension (HT) is well-known to place patients at risk for heart disease and often coexists with diabetes mellitus. Persistent HT increases the morbidity and mortality risk. Both the parasympathetic and sympathetic nervous systems innervate the heart and control heart rate (HR) (chronotropic activity) and strength of compression (inotropic activity). Only the SNS innervates the vasculature, thereby controlling peripheral resistance, and mediates the baroreceptor reflex (BRR), which in turn mediates blood pressure (BP). The angiotensin-renin system controls fluid levels in the body, including blood volume. Angiotensin directly affects the SNS, and the SNS indirectly affects angiotensin [26]. Conditions that result in increases in sympathetic activity may lead to chronic increases in BP and ultimately HT. A BP regulation abnormality related to CAN is due to the deterioration of its circadian rhythm. Decreases in parasympathetic tone at night lead to sympathetic prevalence and result in a lack of or less than 10% reduction in nocturnal blood pressure. Some patients with significant sympathetic predominance have a rise in blood pressure during the night in comparison with daytime blood pressure—this phenomenon is called “reverse dipping.” Such “nondipper” or “reverse-dipper” CAN subjects more often experience left ventricular hypertrophy and cardiovascular events [27].

A frequent clinical manifestation of CAN is orthostatic hypotension. This phenomenon is defined as a decrease in systolic blood pressure > 20 mmHg (or >30 mmHg in hypertensive patients) or diastolic blood pressure > 10 mmHg when changing body position from supine to standing [21]. A change from lying to standing normally results in activation of a baroreceptor-initiated, centrally-mediated sympathetic reflex, resulting in an increase in peripheral vascular resistance and cardiac acceleration. In patients with diabetes, orthostatic hypotension is usually attributable to damage to the efferent sympathetic vasomotor fibers, particularly in the splanchnic vasculature [21]. Orthostatic hypotension causes many symptoms such as lightheadedness, dizziness, faintness, presyncope, and syncope. Orthostatic hypertension may make hypertension treatment difficult.

Also, there are clinical data indicating that CAN is associated with arterial stiffness [28–30]. Arterial stiffness leads to an increase in systolic blood pressure because the heart ejecting into a stiffening arterial bed must generate higher end-systolic pressures for the same net stroke volume. This leads to increased decay of arterial pressure and volume during systole, causing a reduced arterial volume at the onset of diastole, which in turn causes a fall in diastolic blood pressure. The direct clinical consequences of increased arterial stiffness are increased risk of stroke as a result of increased systolic blood pressure, left ventricular hypertrophy as a result of increased cardiac afterload, and decreased coronary perfusion owing to the decrease in diastolic blood pressure [31].

### 3.3. Heart Failure

CAN can lead to abnormalities in the left ventricular systolic and predominantly diastolic function. Echocardiographic studies showed that CAN is significantly associated with reduction in the peak diastolic filling and an increase in the atrial component of diastole [32]. Also, MRI showed that CAN is associated with increased LV mass and concentric remodeling as assessed by MRI independent of age, sex, and other factors [33]. However, abnormalities other than CAN in patients with DM, such as interstitial myocardial fibrosis and microangiopathic or metabolic changes, may also be responsible for left ventricular dysfunction. On the other hand, the parasympathetic denervation observed in the early stages of CAN leads to dominant sympathetic tone promoting metabolic changes, including high myocardial catecholamine levels [9]. This catecholamine rise has been reported to induce mitochondrial uncoupling, switching energy generation at cardiac level from glucose to free fatty acids and therefore increasing oxygen demand, and finally leading to hypertrophy and left ventricular remodeling. Alterations at the biochemical and cellular level lead to programmed cell death and fibrosis [34]. The result of these changes can be clinically present as heart failure, mainly with preserved left ventricular systolic function (diastolic heart failure), which is also related to high morbidity and mortality rates.

### 3.4. Arrhythmias

The ANS has an important influence on heart rhythm. The sinus node is innervated by the PNS and SNS, and the balance between these systems is important for control of HR.

Inappropriate sinus tachycardia is a common manifestation of CAN that occurs at a relatively early stage of the disease. An HR > 90 bpm can be observed as a result of parasympathetic withdrawal. A fixed HR without changes during sleep, exercise, or stress is a sign of complete cardiac denervation. An impaired HR response to exercise leads to exercise intolerance [32].

The arrhythmia typically associated with changes of the ANS is atrial fibrillation (AF). In the 1990s, Phillipe Coumel stated that AF is not a homogeneous entity, and many factors are responsible for a number of different behaviors. He observed that the electrophysiological characteristics of atrial cells (action potential duration and refractoriness and conduction speed) are modulated differently by parasympathetic and sympathetic influences. Parasympathetic effects tend to favor macroreentry phenomena, whereas sympathetic ones favor abnormal automaticity and triggered activity [35]. In normal hearts, vagal influences are predominant, thus explaining why the clinical pattern of vagal-mediated paroxysmal AF is preferentially observed in the absence of detectable heart disease, in young male adults, with an ECG pattern of common flutter alternating with fibrillation. Sympathetically mediated AF is observed in the presence of any heart disease, the first effect of which is to provoke a vagal withdrawal. This clinical situation can be observed as a result of CAN, even in relatively early stages. In the group of DM patients with CAN, a higher number of recurrences of paroxysmal AF are observed in comparison to DM without CAN (47 episodes per year versus 22 episodes per year, resp., *p* < 0.01). This study also revealed that the presence of CAN caused a significant increase in P-wave duration and dispersion. That inhomogeneous atrial depolarization is the potential trigger of paroxysmal AF in patients with CAN [36].

The influence of CAN on nonsustained ventricular arrhythmias, according to our knowledge, is not well-documented. However, life-threatening ventricular arrhythmias and sudden cardiac deaths are clearly associated with CAN [5, 7, 8].

### 3.5. Mortality and Sudden Cardiac Death

CAN is associated with an increased total and cardiovascular mortality. In a meta-analysis of 15 studies, Maser et al. [37] found that the pooled-estimated relative mortality risk was 2.14 (95% CI: 1.83–2.51, *p* < 0.0001) for CAN patients. The magnitude of the association was stronger for those studies for which two or more measures were used to define CAN. The pooled relative risk for studies that defined CAN with the presence of two or more abnormalities was 3.45 (95% CI 2.66–4.47; *p* < 0.001) compared with 1.20 (1.02–1.41; *p* = 0.03) for studies that used one measure. CAN also had the strongest association with mortality among other risk factors in the EURODIAB IDMM Complications Study [38]. In the population of the ACCORD trial, which included patients with high risk of cardiovascular event, CAN was an independent risk factor of all-cause mortality (HR 2.14, 95% CI: 1.37–3.37) and cardiovascular mortality (HR 2.62, 95% CI: 1.4–4.91) after a mean follow-up of 3.5 years [13].

CAN is also associated with a higher risk of malignant ventricular arrhythmias and sudden death [39]. On the one hand, severe silent ischemia or myocardial infarction can induce life-threatening arrhythmia and sudden death. Additionally, lethal arrhythmias can be explained directly by an imbalance between the sympathetic and parasympathetic parts of the ANS. The EURODIAB IDDM Complication Study showed the association between CAN and QT prolongation [40]. Other studies also confirmed the influence of CAN on QT prolongation [41, 42]. It has been postulated that QT prolongation predisposes to cardiac arrhythmias and sudden death.

Other mechanisms depending on autonomic imbalance, such as impaired response to hypoxic state, reduced hypoglycemia awareness, and prolonged hypoglycemia episodes, can also be responsible for malignant ventricular arrhythmias and finally lead to sudden death [43, 44].

## 4. Diagnosis of CAN

Traditionally, the diagnostic methods of cardiac autonomic neuropathy are based on heart rate variability (HRV) and changes in BP during certain physiological maneuvers. In the 1970s, Ewing et al. [45] proposed five simple tests to measure autonomic functions. These test are (1) the R-R alterations to paced deep breathing (expiration : inspiration ratio and E : I ratio); (2) HR response to standing—30 : 15 ratio—which is the ratio of the longest R-R interval (between the 20th and 40th beat) to the shortest interval (between the 5th and 25th beat) elicited by a position change from horizontal to vertical; (3) the HR response during the Valsalva maneuver; (4) the BP response to standing; and (5) the BP response to sustained handgrip caused by the muscle contraction using a handgrip dynamometer (Table 2).

The first two tests measure parasympathetic function—mainly the ability of the vagal nerve to slow the HR during procedures which increase HR. The Valsalva maneuver primarily represents also parasympathetic activity, but autonomic changes also include a sympathetic component. The last two tests show changes in sympathetic function and involve baroreflex-mediated blood presser fluctuations. The American Diabetes Association recommends the use of these Ewing's tests in the diagnosis of CAN [46].

According to the Toronto Diabetic Neuropathy Expert Group, the most widely used tests assessing cardiac parasympathetic function are based on the time-domain HR response to deep breathing, a Valsalva maneuver, and postural change. Of these tests, HR to deep breathing has the greatest specificity (∼80%). Cardiovascular sympathetic function is assessed by measuring the BP response to orthostatic change and a Valsalva maneuver [47]. The experts did not recommend the handgrip test, but this test is still used in clinical studies [48].

The short-term ECG recordings can be analyzed by dedicated software in the frequency domain. This method usually uses the Fourier method, which transforms R-R intervals into waves with three basic components: very low frequency ≤ 0.04 Hz (VLF), low frequency 0.04–0.15 Hz (LF), and high frequency 0.15–0.4 Hz (HF). HF represents vagal activity, whereas LF combines the effect of sympathetic and parasympathetic influence. A decrease in HF is a sign of parasympathetic dysfunction, in the early stages of autonomic dysfunction in diabetes, when sympathetic predominance is observed it leads to an increase in LF/HF [49].

It is not clear if classical Ewing's tests or time-domain methods are better for diagnosis of CAN. Studies comparing the two methods showed high—over 80%—correlations between their results [50]. However, Ewing's tests are simpler and can be more easily implemented during routine clinical use.

Another diagnostic method of CAN can be based on HRV assessed during classic 24 h Holter ECG monitoring and use of statistical indexes in the time and frequency domain. It is obvious that reduction in HRV is associated with CAN, but this method has no standard values for the diagnosis of CAN [51]. Also, during 24 h recording, many factors can have an influence on HRV parameters, such as concomitant illness, use of medication, and lifestyle factors (exercise, stress, smoking, etc.). The literature has been described in detail regarding HRV analysis based on Holter ECG, but it is beyond the scope of this article [49].

The baroreceptor reflex (BRR) is another method that can be used for detecting CAN. In the physiological BRR, an increase of BP induces an increase in the vagal cardiac efferent and reduction in the sympathetic activity, resulting in bradycardia, hypotension, and peripheral vasodilatation [52]. A reduction in BP induces opposite responses. The BRR test can be used for detecting CAN and correlates very well with the classical Ewing's tests [53]. Studies on patients with DM have concluded that an impaired BRR is a strong independent risk factor for mortality [54].

Another Holter-based technique for evaluating CAN is heart rate turbulence (HRT). HRT is an indirect measurement of baroreflex sensitivity and refers to sinus rhythm fluctuations following premature ventricular beat. Physiologically, after ventricular beat, sinus rate acceleration and next deceleration are observed. There are two components of HRT—turbulence onset (TO) and turbulence slope (TS). The initial heart rhythm acceleration after ventricular premature beat is defined as TO and the ensuring deceleration as TS [55]. HRT parameters could be a useful monitoring tool for the function of the autonomic nervous system in patients with diabetes mellitus [56]. Balcıoğlu et al. [57] revealed that decreased TS has a correlation with CAN severity. Although HRT tests are not standardized for detection of CAN and have no cutoff values for diagnosis of CAN, TS < 3.32 ms/R-R was 97% sensitive and 71% specific for diagnosis of CAN as detected by Ewing's tests [57]. The major limitation concerning the measurement of HRT is that the presence of ventricular premature beats is mandatory for its evaluation.

Other methods currently used in the detection of CAN are single photon emission computed tomography (SPECT) and positron emission tomography (PET) with sympathetic neurotransmitter analogs such as ^123^I-metaiodobenzylguanidine (^123^I-MIBG), ^11^C-metahydroxyephedrine (^11^C-HED), and ^11^C-epinephrine [9].

The lack of standardization, the high cost, and the requirement of specific equipment and skilled staff restrict the role of scintigraphy as a research tool, and it is not a part of everyday clinical practice.

According to the latest Standards of Medical Care in Diabetes published by the American Diabetes Association, symptoms and signs of autonomic neuropathy should be assessed in patients with microvascular and neuropathic complications. CAN may be completely asymptomatic and detected only by decreased heart rate variability with deep breathing. Major clinical manifestations of CAN include resting tachycardia (>100 bpm) and orthostatic hypotension (a fall in systolic or diastolic blood pressure by >20 mmHg or >10 mmHg, resp., upon standing without an appropriate increase in heart rate) [58].

## 5. Stages and Diagnostic Criteria for CAN

Ewing's tests (Table 2) are the gold standard clinical tests for the diagnosis of CAN. The reference values for an abnormal test are age-dependent. According to the CAN Subcommittee of the Toronto Consensus Panel [52], only one abnormal test is sufficient to diagnose possible or early CAN. Two or more abnormal tests indicate definite CAN. The presence of orthostatic hypotension in addition to abnormal tests signifies severe CAN [52].

**Figure 1:**
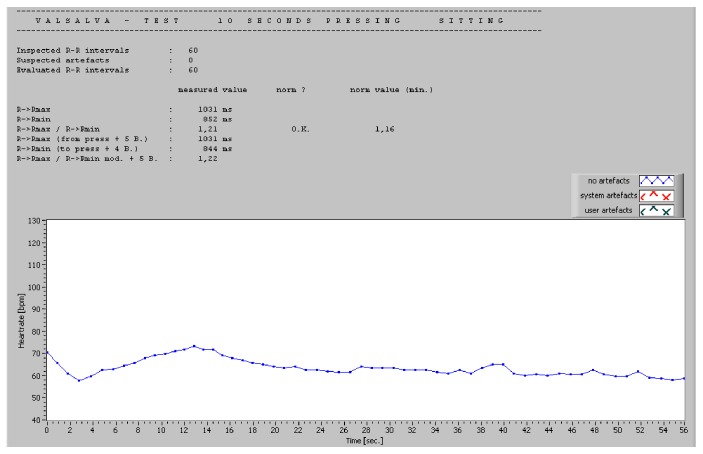
Example of normal Valsalva test.

**Figure 2:**
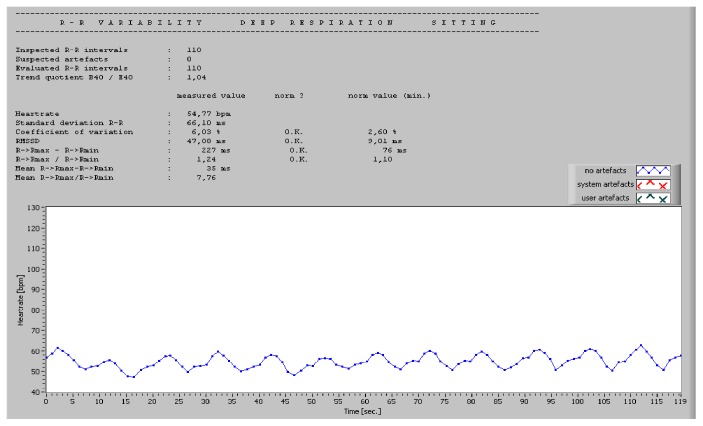
Example of normal deep respiration test.

For bedside testing, a dedicated software can be used. The authors have personal experience with ProSciCard III (MEWICON CATEEM-Tec GmbH, Germany), which offers the possibility of an online ECG measurement and a subsequent offline detailed analysis with comparison to normal values. The tests included in the software are RR variability at rest (over 170 intervals, 5, 10, or 30 min), RR variability during deep breathing, Valsalva test (10 or 15 s), and orthostasis test. The results are not only typical Ewing's parameters but also time and frequency domain HRV during each test. Examples of normal tests are presented in Figures 1 and 2.

## 6. Conclusions

CAN is a frequent chronic complication of DM with potentially life-threatening outcomes. Although there are available simple bedside tests for diagnosis of CAN, it is often overlooked. The statements of the Toronto Diabetic Neuropathy Experts Group recommended screening for all diabetic patients [52]. Because CAN has a significant negative influence on coexisting heart diseases and the most common methods used for the diagnosis of CAN are based on ECG, cardiologists should also be responsible for diagnosis of CAN.

## Figures and Tables

**Table 1:** Prevalence of cardiac autonomic neuropathy in different studies.

Reference	Year	*N* of subjects	Type of DM	Population	Diagnosis tests for CAN	Criteria applied	CAN prevalence (%)
O'Brien [44]	1991	506	Type 1	Mean age 45 years, mean DM duration 15 years	HRV: rest, deep breathing, Valsalva, lying to standing	2 or more test positive	17

Ziegler [59]	1992	647 524	Type 1		HRV: coefficient of variations, spectral analysis, Valsalva, lying to standing	3 or more test positive	25.3
		524	Type 2				34.3

Kennedy [60]	1995	290	Type 1	Potential recipients of a pancreas transplant	Deep breathing	Single test positive	90
					Valsalva		88

Kempler [12]	2002	3007	Type 1	Mean age 32 years, mean DM duration 14 years	HR lying to standing, postural BP	Single test positive	36

Gaede [2]	2003	160	Type 2	Mean age 55 years, HbA1c 8.8% at baseline	Deep breathing, postural BP	Single test positive	27.5

Low [61]	2004	83	Type 1		Sudomotor axon-reflex test, Valsalva, BP and HR response to standing, deep breathing	2 or more test positive	54
		148	Type 2				73

Pop-Busui [5]	2010	620	Type 1—intensive treatment group	Mean age 47 years, mean DM duration 26 years	Deep breathing, Valsalva, postural BP	2 or more test positive	29
		591	Type 1—conventional treatment group				35

DM: diabetes mellitus; CAN: cardiac autonomic neuropathy; HRV: heart rate variability; BP: blood pressure; HR: heart rate.

**Table 2:** Ewing's tests and CAN diagnosis.

Test	Description
Expiration/inspiration (E/I) ratio	The patient was asked to take deep breaths for 10 minutes with frequency about 6 breaths/min.
Valsalva maneuver	The patient was asked to blow into the special manometer to maintain the pressure at about 40 mmHg for 15 s.
Postural heart rate response: maximum-minimum (30 : 15 ratio)	Heart rate was measured in the horizontal position and again two minutes later after standing upright.
Postural blood pressure response	Blood pressure was measured in the horizontal position and after 1, 3, and 5 minutes after standing upright.
Isometric handgrip test	The patient was asked to grip the dynamometer for 5 minutes.
